# Phototherapy^☆^^☆☆^

**DOI:** 10.1016/j.abd.2021.03.001

**Published:** 2021-04-02

**Authors:** Norami de Moura Barros, Lissiê Lunardi Sbroglio, Maria de Oliveira Buffara, Jessica Lana Conceição e Silva Baka, Allen de Souza Pessoa, Luna Azulay-Abulafia

**Affiliations:** Department of Dermatology, Hospital Universitário Pedro Ernesto, Universidade do Estado do Rio de Janeiro, Rio de Janeiro, RJ, Brazil

**Keywords:** Phototherapy, PUVA therapy, Ultraviolet therapy

## Abstract

Of all the therapeutic options available in Dermatology, few of them have the history, effectiveness, and safety of phototherapy. Heliotherapy, NB-UVB, PUVA, and UVA1 are currently the most common types of phototherapy used. Although psoriasis is the most frequent indication, it is used for atopic dermatitis, vitiligo, cutaneous T-cell lymphoma, and cutaneous sclerosis, among others. Before indicating phototherapy, a complete patient assessment should be performed. Possible contraindications should be actively searched for and it is essential to assess whether the patient can come to the treatment center at least twice a week. One of the main method limitations is the difficulty that patients have to attend the sessions. This therapy usually occurs in association with other treatments: topical or systemic medications. Maintaining the regular monitoring of the patient is essential to identify and treat possible adverse effects. Phototherapy is recognized for its benefits and should be considered whenever possible.

## Introduction

Phototherapy consists of the therapeutic use of ultraviolet (UV) radiation. It can be performed with exposure to sunlight, ultraviolet A (UVA) or ultraviolet B (UVB) radiation. The wavelengths administered and the UV radiation doses vary according to the proposed indication.^1^

Ultraviolet radiation (UVR) encompasses wavelengths ranging from 200 to 400 nm.

It is divided into:

UVA (320–400 nm), which is subdivided into UVA2 (320–340 nm) and UVA1 (340–400 nm).

UVB, subdivided into broadband UVB (290–320 nm) and narrowband UVB (NB-UVB), from 311 to 313 nm.^2^

UVC (200–290 nm), which is blocked by the ozone layer and by the oxygen of the atmosphere and which is not used for phototherapy.^1^

The benefits of phototherapy have been recognized since the 20^th^ century BCE. Although psoriasis is the most frequent indication, phototherapy has been used successfully in several other dermatoses, such as atopic dermatitis, vitiligo, cutaneous T-cell lymphoma, and cutaneous sclerosis, among others. Using controlled and repeated UV exposures, it is possible to induce regression or control the evolution of these dermatoses.^3^

Most of the time, phototherapy is used in combination with topical or systemic medications for better disease control.^4^

Just like any other therapy, it has side effects. Most of the time, they are acute and transient, including erythema and burns, and attention should be paid to possible adverse events during treatment.^4^

Accessibility to the phototherapy unit is an important limiting factor for undergoing this type of treatment, despite the degree of satisfaction reported by users.^5^

## History

For many centuries, sunlight treatment or heliotherapy has been instituted for the treatment of skin diseases. In Egypt and India, 3,500 years ago, people had the habit of using plant extracts or seeds, with subsequent exposure to the sun for the treatment of skin diseases.^6^

In the 19^th^ century, the modern era of the use of light started. Downes and Blunt, in 1877, published results of research in which exposure to light inhibited fungal and bacterial growth.^7^

In the 20^th^ century, phototherapy was recognized as a medical science after Niels Finsen received the Nobel Prize of Medicine in 1903. Twenty years later, William Henry Goeckerman started using a lamp that emitted mainly UVB, together with coal tar to treat psoriasis. This treatment became very popular and was used for decades.^6^, ^7^

The increase in the effectiveness of phototherapy started in 1947, with the isolation of 8-Methoxypsoralen (8-MOP) and 5-Methoxypsoralen (5-MOP), derived from the Ammi Majus Linn flower.^7^

There are reports on the use of this plant that dates back to the 13^th^ century, when the Arab physician Ibnal-Bitar mentioned in his book “Mofradat El-Adwiya” the effects of ingesting Ammi Majus extracts, followed by exposure to sunlight, for vitiligo repigmentation. This treatment was the oldest form of what is currently called photochemotherapy, a modality defined as the ingestion of a psoralen followed by exposure to UVA (320–400 nm). In 1974, the term PUVA (Psoralen-ultraviolet A) was created by Thomas B. Fitzpatrick and John Parrish to name this therapeutic modality.^7^

The development of photochemotherapy with PUVA paved the way for the research into new modalities. NB-UVB radiation (311–313 nm) was discovered in 1988, gradually replacing broadband UVB (290–320 nm).^6^

Phototherapy started being used in Brazil in the 1980s. It was also in this decade that a new type of phototherapy was introduced, extracorporeal photochemotherapy, initially for the treatment of cutaneous erythrodermic T-cell lymphoma.^8^

A major advance in the field of phototherapy was the development of UVA1 lamps (340–400 nm), which occurred in the early 1990s. Used mainly for the treatment of atopic dermatitis and scleroderma, this modality of treatment does not require the use of psoralen, thanks to its greater penetrating power.^9^

More recently, in 1997, phototherapy with an excimer laser (UVB - 308 nm), a subtype of NB-UVB, was introduced for the treatment of psoriasis and is currently used in other diseases, such as vitiligo.^2^, ^6^

From the heliotherapy practiced in Ancient Egypt to the development of the excimer laser, phototherapy has been part of the therapeutic arsenal of Dermatology, establishing its importance in clinical practice.

## Mechanism of action

UVR is absorbed by the chromophores (molecules that have the capacity to absorb certain wavelengths), such as DNA, nucleotides, lipids, amino acids, trans-urocanic acid, and melanin. UVR causes changes in the structure and function of chromophores. The molecules thus modified are called photoproducts, which participate in apoptosis, inflammation, immunosuppression, and photocarcinogenesis.^1^

The depth reached in the skin by each radiation type depends directly on its wavelength. UVB radiation (broadband and NB-UVB) has a shorter length, being absorbed by the epidermis and the superficial portion of the dermis. The UVA waves (1 and 2) have a longer length, penetrating up to the dermis.^1^

Both UVA (PUVA or UVA1) and UVB (broadband and NB-UVB) have immunosuppressive and antiproliferative effects.^1^

The reduction in the number of macrophages, the inhibition of inflammatory cytokine production (IL-2, IL-8, IL-9, IL-17, IL-22, IL-23, TNF-α, and IFN-γ) and IL-10 (immunosuppressive cytokine) induction can occur in both the UVR spectra and contribute to the anti-inflammatory effect of phototherapy.^1^

Although there are still gaps in the knowledge about the UVR mechanisms of action, one can didactically separate the most characteristic effects of UVB (broadband and NB-UVB) and UVA, subdivided in this topic into PUVA and UVA1:

### UVB (broadband, NB-UVB and excimer laser)

- Induces the apoptosis of keratinocytes and T-cells through direct molecular alteration of DNA and, therefore, causes the inhibition of its transcription and interrupts the cell cycle.
- Promotes the conversion of trans-urocanic acid (trans-UCA) into cis-urocanic acid (cis-UCA), which leads to inhibition of contact hypersensitivity, in addition to impairing the function and reducing the amount of antigen-presenting cells.
- Reduces the number of natural killer cells.
- Intensifies the production of reactive oxygen species, leading to increased synthesis and enzyme activity of the cellular antioxidant system, which modifies the immune reaction pattern.^1^, ^3^

### UVA (PUVA and UVA1)

The UVA wavelength is not so easily absorbed by the DNA molecule. It acts mainly through other chromophores, generating free radicals (reactive oxygen species), which cause indirect damage to the genetic material, promoting DNA degradation.^9^

### PUVA

- Always performed after the administration of psoralens, furocoumarin compounds that act as chromophores for UVA. After exposure to UVA, they absorb photons, become activated and covalently bind to the DNA bases. Thus, they form cross-linked pairs, which have an antiproliferative, antiangiogenic and apoptotic effect.^3^
- Stimulates melanogenesis, although the mechanism of action is not known.^3^
- Induces the apoptosis of T cells infiltrated into the skin.^3^
- Induces the expression of collagenase-1 in dermal fibroblasts.^3^
- Reduces the synthesis of collagen I and III, leading to an antifibrotic effect.^3^

### UVA1

- Prevents direct damage to the DNA, as it has the lowest energy within the UV spectrum.^9^
- Induces apoptosis of lymphocytes, mast cells and Langerhans cells.^9^
- Inhibits the expression of cytokines associated with Th2 response, such as IL-5, IL-13 and IL-31.^9^
- Reduces collagen and hydroxyproline levels, proportionally to the utilized dose.^10^
- Activates collagenases, which participate in the breakdown of dermal collagen.^9^
- Changes the quality of collagen, reducing its density.^10^
- Inhibits fibroblast activity.^10^

In scleroderma, it can induce neovascularization and decrease apoptosis of endothelial cells. This factor, associated with the other above mentioned mechanisms of action, makes UVA1 to be frequently prescribed for sclerosing skin diseases.^10^

It is important to note that the effects of UVR on the human body do not change abruptly from one spectrum to another. In fact, these effects are continually changing from one wavelength to another and can even add up.^9^

## Types of phototherapy

### UVA

UVA rays (320–400 nm) are subdivided into:

UVA1 (340–400 nm) reaches the epidermis, the middle and deep dermal components, especially blood vessels.^3^

UVA2 (320–340 nm) resembles UVB, with more superficial penetration.^3^

### PUVA

Before the development of UVA1 lamps, the UVA phototherapy that was in use was PUVA, a method that by definition requires the use of psoralens. Psoralen is a photosensitizing substance that can be used systemically via the oral route (capsule) or topically. The latter route employs psoralen in cold cream, alcoholic solution, emulsion, or diluted (in a full or partial bath).^4^ For patients with gastric intolerance, there is the possibility of the systemic use of psoralen through rectal administration (suppository).^11^

Treatment with systemic PUVA (oral or rectal administration) involves the use of methoxypsoralen two hours before exposure to UVA radiation, usually performed 2 to 3 times a week. The radiation dose is progressively increased until a mild erythematous reaction occurs. After the session, it is necessary to maintain skin and eye photoprotection for 24 hours.^11^, ^12^

Topical PUVA therapy (applying psoralen in cold cream, solution or emulsion to the lesions only) is an option in case of localized dermatoses. This type of administration, while less practical for the patient, prevents the gastrointestinal side effects of oral medication.^12^

### PUVA Bath

PUVA bath is a topical phototherapy as effective as oral PUVA therapy. It is a good option for patients with extensive injuries, but with contraindications for systemic therapy. The technique consists of exposure to UVA radiation after the patient has bathed in a bathtub containing 100 liters of warm water and 37.5 mL of 1% 8-methoxypsoralen.^13^ 8-MOP is more soluble in water, allowing the phototoxic effect to quickly disappear after the treatment, with rinsing in running water, without the need to use photoprotection measures after the session.^14^ The PUVA bath is mainly indicated for moderate to severe plaque psoriasis and chronic dermatoses of the palmoplantar region.^13^

### UVA1

Phototherapy with UVA1, unlike PUVA, omits UVA2 and does not require the use of psoralens. ^4^

It is divided into 3 energy ranges:

- Low dose: 10–20 J/cm^2^
- Intermediate dose >20–70 J/cm^2^
- High dose >70–130 J/cm^2^.^4^

This modality was seldom used by most Dermatology departments worldwide, as it implied high heat emission and prolonged time of exposure.^1^, ^4^ The lamps were made of high-emission metal halides (Sellamed 4000 W, Sellas Medizinische Geräte GmbH, Ennepetal, Germany), which were not available in Brazil. They emitted high doses of energy (130 J/cm² in a single dose), are now in disuse.^4^

Currently, in Brazil, there is a type of UVA-1 lamp that alleviates these disadvantages. It is the UVA-1 fluorescent lamp, marketed by Philips (TL10R 100 W, Philips).

UVA1 application has protocols that change according to the disease to be treated, but treatment is usually performed 3 to 5 times a week, with doses starting between 20–30 J/cm^2^, with progressive increase.^12^

The time of exposure during the session is calculated by the ratio between the number of Joules and the emission power of the lamp, assessed by the radiometer in mW. As an example, considering that the current UVA1 lamps emit 20 mW, to calculate the patient's exposure time, receiving 0.5 J/cm², we first transform 0.5 J into 500 mJ and then divide the desired dose of 500 mJ by 20 mW, which results in 25 seconds.^15^

The use of the radiometer is essential, as it says how many mW the lamp emits. As the lamps lose their emission capacity over time, this implies a dose adjustment and an increase in the time of exposure, requiring the periodical substitution of the lamps.^15^ This is valid for all types of phototherapy.

UVA1 is a good option for the treatment of inflammatory and autoimmune diseases. The treatment can be carried out exclusively through this modality or in combination with conventional therapies. It can be carried out on children, pregnant women and patients with contraindications to the use of psoralens (not employed in this modality).^12^

UVA1 has fewer adverse effects than PUVA, as it omits UVA2 which, like UVB, has the ability to cause erythema and carcinogenesis.^3^

### UVB

UVB radiation corresponds to wavelengths between 290 and 320 nm. It is divided into broadband UVB (290–320 nm) and NB-UVB (311–313 nm). It is indicated for psoriasis, atopic dermatitis, renal and hepatic pruritus, parapsoriasis, mycosis fungoides, vitiligo, acute and chronic graft-versus-host disease, among others. As it does not involve the administration of psoralens, it can be indicated for children and pregnant women.^4^, ^16^, ^17^

Currently, broadband UVB is in disuse. Most centers use NB-UVB, which is more effective than broadband UVB, mainly in the treatment of psoriasis, atopic dermatitis and vitiligo, with less potential to generate adverse events.^4^, ^18^ The NB-UVB dose that can cause hyperplasia, edema, burning and the depletion of Langerhans cells is 5 to 10-fold higher than the broadband UVB dose.^16^

Treatment with UVB (broadband and NB-UVB) can be applied 3 to 6 times a week.^4^ However, in most centers, it is performed 2 to 3 times a week.

There are two ways to determine the initial radiation dose:

1 Determination of the Minimum Erythematous Dose (MED): the minimum amount of irradiation necessary to cause erythema. The therapy is started with 70% of the MED. This method is in disuse due to practical limitations. ^4^
2 Beginning the therapy with a standard radiation dose according to the patient's phototype. This method is currently the most widely used. ^4^

After defining the initial dose, every one or two sessions, the radiation dose is increased by 10% to 30% until there is asymptomatic erythema. The peak of the erythematous reaction occurs between 12 and 24 hours after radiation exposure. Eye protection is essential, but only during the phototherapy session.^4^, ^16^

In the event of disease recurrence or worsening, the frequency of treatment should be increased and, in some cases, the dose should be elevated, according to each patient’s tolerance. Upon reaching remission, maintenance therapy is generally not indicated, with the exception of mycosis fungoides, which may require prolonged treatment to maintain disease control.^4^

## Other types of phototherapy

### Excimer laser and lamp

This phototherapy model was introduced in the therapeutic arsenal of Dermatology in 1997. As a subtype of NB-UVB, with a wavelength of 308 nm, it was approved for the treatment of psoriasis, atopic dermatitis and vitiligo in the United States. It is effective for several other localized (less than 10% of body surface) and chronic inflammatory dermatoses. It can be performed in places that are difficult to access with traditional phototherapies, such as the scalp, and palmoplantar skin.^2^

Laser phototherapy is directed to the lesion through a tip with a spot measuring 14 to 30 mm in diameter, sparing healthy skin. This characteristic allows higher doses to be administered from the beginning. Therefore, fewer adjuvant treatments are needed and long-term side effects are reduced.^2^ Its emission depends on a mixture of xenon and chloride gas, which form unstable “excited dimers”(excimer). When dissociated, these dimers produce a coherent wavelength of 308 nm, which penetrates primarily the epidermal cells and, secondarily, into fibroblasts.^19^

The excimer lamp, on the other hand, emits inconsistent light and, consequently, requires a longer time than the laser to emit the same fluency. As advantages, it allows the treatment of more extensive areas, with lower operational costs, as well as being easier to transport.^20^

Both the excimer laser and the excimer lamp have shown similar or superior effectiveness to NB-UVB in the treatment of psoriasis and vitiligo. In atopic dermatitis, despite promising results in relation to pruritus improvement, the European and American guidelines do not endorse its use, due to the scarce number of studies.^20^, ^21^

More recently, the role of the excimer laser in the treatment of alopecia areata has been investigated. The results are promising and the absence of significant side effects, especially when compared to traditional therapies (corticotherapy and topical immunotherapy), encourages its use. Further studies are still necessary to determine whether the excimer lamp would have the same effectiveness as the excimer laser in this usage.^22^

### Ultraviolet “combs”

They are mainly indicated for the treatment of scalp psoriasis. Patients with seborrheic dermatitis also benefit from this therapy. This method allows the direct application of light to the scalp. The accessories are removable and similar to combs, easy to sterilize.^23^

Most devices emit NB-UVB and although scientific studies are lacking on their therapeutic effectiveness, there have been no reports of acute or chronic side effects after the adequate use of the method.^23^

### Home treatment

Home phototherapy with UVB can be prescribed for selected patients, who show adequate cognition and treatment adherence. However, worldwide, some factors negatively influence on the prescription of this therapy, such as difficulty in controlling the equipment, as well as the duration of sessions performed by the patient, in addition to the lack of an adequate reimbursement system.^24^

Less conventional phototherapy methods, such as heliotherapy (exposure to sunlight), with or without psoralen, have been recommended in situations when conventional phototherapy is not feasible for the patient.^25^

### Clinical and laboratory tests prior to phototherapy

Before choosing the phototherapy type, a complete assessment of the patient is essential. Dermatological examination of the entire integument should be performed to assess the dermatosis severity and extent, determine the phototype and the degree of photodamage. It is also important to describe in the patient’s record the aspect for any nevi he presents at the examination and also detect premalignant or malignant skin lesions.^26^

A previous examination of the eyes of the patient is essential. If an abnormality is detected, the follow-up should be performed at least once a year with an ophthalmologist.^26^, ^27^

The laboratory tests that should be requested for this phototherapy modality include kidney and liver function, in addition to beta-HCG to rule out any pregnancy. In the case of concomitant therapy with retinoids, a lipid profile should be requested.^24^

The request for the ANF test is debatable. If there is a family history of or suspected collagen disease, it is advisable to request it. Otherwise, it is not part of the previous exams for phototherapy.

## Indications

### Psoriasis

Psoriasis is the disease that is most commonly treated with phototherapy. In addition to being effective, phototherapy is considered a safe option. It is usually indicated when topical treatments do not show good results or are not practical for the patient, such as those with extensive psoriasis. It is the only viable therapeutic option in cases of severe psoriasis affecting individuals with contraindications for systemic treatments.^28^

Currently, NB-UVB is the therapeutic modality of choice. Studies have shown its greater effectiveness compared to broadband UVB.^17^ Regarding UVA1, further studies are needed to compare its effectiveness with other types of phototherapy, given the small number of patients included in the studies done so far.^29^

NB-UVB is considered the first-choice treatment for pregnant women with extensive disease. It can be used with caution in children, but it is not the first choice, as the possible carcinogenic potential and anxiety in young children are limiting factors for this group.^16^

The excimer laser/lamp is useful in the treatment of lesions affecting less than 10% of the body surface, such as palms, soles, elbows and knees.^28^ It has the same effectiveness as PUVA for the treatment of non-pustular palmoplantar psoriasis.^30^

PUVA can be used topically or systemically, being indicated for stable plaque psoriasis. Despite being highly effective, it has a worse tolerance profile than NB-UVB and there is greater evidence of carcinogenic potential, therefore, it is considered a second-line option for psoriasis treatment.^16^ In some cases, phototherapy can be combined with oral retinoids, reducing treatment time.^30^

The mechanism of action of phototherapy in the treatment of psoriasis is not completely understood. UVB (broadband and NB) is known to induce apoptosis of pathogenic T lymphocytes and keratinocytes, leading to reduced epidermal hyperproliferation and local and systemic immunosuppression.^28^, ^30^

NB-UVB inhibits the Th17 pathway, which is crucial for disease pathogenesis. Moreover, it increases stability and restores regulatory T-cell function. Accumulated doses of this modality are believed to reduce levels of plasmin, a potent inflammatory activator, contributing to its therapeutic effect.^28^, ^30^

It is believed that UVA1 induces T-cell apoptosis and reduces inflammatory cytokine levels, such as TNF-α and INF-γ. Additionally, UVA1 has been shown to inhibit the activity of antigen-presenting cells and to reduce the amount of Langerhans cells in the epidermis.^31^

The treatment should be discontinued when complete disease remission is achieved or if there is no response. Phototherapy provides high rates of patient satisfaction.^18^

The duration of remission correlates with the reduction in Psoriasis Area and Severity Index (PASI) at the end of treatment. PASI reduction is on average 70% when NB-UVB is applied and 80% when treated with PUVA, results comparable to those seen with immunobiological drugs.^28^

### Vitiligo

Vitiligo is an acquired pigmentation disorder, characterized by the loss of epidermal melanocytes. In most cases, it behaves in a chronic and stable manner, with short periods of progression.^32^

NB-UVB and PUVA phototherapy constitute the main treatment modalities for this dermatosis. Currently, NB-UVB is the first-line treatment for the generalized form. For localized disease, the excimer laser and the excimer lamp are more adequate.^33^

Yones et al. demonstrated the superiority of NB-UVB phototherapy over PUVA in a randomized clinical trial. In that study, patients treated with NB-UVB had a 50% higher repigmentation rate than patients treated with PUVA after six months of follow-up.^33^

In addition to its superior effectiveness, treatment with NB-UVB has other advantages over PUVA: the lack of a photosensitizer, lower cumulative dose and fewer adverse effects.^34^

Nevertheless, phototherapy with NB-UVB does not always bring satisfactory results. Lesions on the face, neck and trunk are more sensitive to phototherapy, while those on the hands, feet, elbows and knees are more resistant, with minimal results. A minimum of six months of treatment is required to assess the patient's response to therapy.^33^

In recent years, several studies on the combination of NB-UVB with topical calcineurin inhibitors or vitamin D analogs have shown good response, suggesting that topical agents can produce synergistic effects when combined with phototherapy, increasing their effectiveness.^34^

### Lymphomas

Cutaneous T-Cell Lymphomas (CTCL) are a heterogeneous group of non-Hodgkin's lymphomas of the skin, with the mycosis fungoides (MF) subtype being the most common variant. Initially, it appears as erythematous patches and plaques and can progress to skin tumors. Extracutaneous involvement is present in some cases.^35^

The United States Cutaneous Lymphoma Consortium recommends phototherapy as a monotherapy regimen for patients with early stages of CTCL/mycosis fungoides (stage IA-IIA), and in combination with systemic therapies for refractory early disease or advanced disease.^36^ Several systemic agents can be safely combined with phototherapy, mainly interferon-alpha and retinoids.^35^

Determining the type of phototherapy to be used, among PUVA, NB-UVB and extracorporeal photochemotherapy will depend on the stage of the disease, the patient's preference and the methods availability. UVA shows better skin penetration than UVB, and patients with thicker plaques, darker skin and folliculotropic T-cell lymphoma may benefit more from PUVA.^36^

The immediate relief that many patients experience due to the decrease in the size and number of lesions, as well as an improvement in pruritus, is significant. ^37^

The phototherapy treatment regimen for CTCL involves 3 phases: induction, consolidation and maintenance.

The first phase may last longer than in other dermatoses treated with phototherapy. The second phase, the consolidation one, lasts from one to three months. This phase can maximize the potential for histopathological and molecular clearance (including loss of the dominant T-cell clone). During the last phase, the maintenance one, the frequency and dose of treatment are kept constant. It is still controversial whether a prolonged maintenance phase after disease remission can reduce recurrence rates, since there is insufficient data for such assertion.^36^

PUVA is the initial choice of phototherapy for CTCL chosen by many specialists. It is effective for early MF, with estimated response rates of 85% for stage IA and 65% for stage IB. Treatment time with PUVA varies from two to four months, with two to three sessions per week.^36^

Despite being in disuse, broadband UVB is a good option for patients with stage IA of the disease and fair skin (phototypes I and II). However, in the hypopigmented variant of MF, the response is limited.^36^

As for the excimer laser, there have been several reports showing the benefits of its use; however, the follow-up was short, being reserved for sites not easily accessible to phototherapy or topical medications, such as acral surfaces or intertriginous areas.^36^

Further studies, with better standardization, are needed to determine the ideal phototherapy regimen, regarding effectiveness and long-term safety.^37^

### Parapsoriasis

Parapsoriasis is a chronic inflammatory skin disorder whose etiology remains unknown.^38^

Previous studies have shown that this disease probably represents different stages of a lymphoproliferative disorder. It has been considered as a separate entity or as the initial form of MF, although this remains debatable.^38^

Skin-targeted therapies are the main therapeutic options for the management of parapsoriasis and early-stage MF.^39^

Phototherapy is indicated for all types of parapsoriasis and its clinical variants. In general, NB-UVB is the preferred treatment modality. PUVA should be used in patients with thick plaques, high phototypes and those not responsive to UVB.^39^

In the case of patients who cannot tolerate or do not respond to PUVA or NB-UVB therapy, low-dose UVA1 therapy seems to be a safe and effective alternative. However, the therapeutic regimen is not established, due to the few studies assessing this therapy.^40^

### Scleroderma

Scleroderma is a chronic connective tissue disease, whose etiology remains unknown. It is characterized by intense collagen deposition in the dermis and, in some cases, in internal organs. The main treatment objective is to increase skin elasticity, improving patient mobility and quality of life, in addition to delaying disease evolution.^10^

As a therapeutic option, phototherapy is safe, as its effect is directed at the skin, without the risk of systemic complications. It represents an effective alternative for individuals who are refractory to topical or systemic treatments. Those with contraindications to immunosuppressive therapy also benefit from the method.^10^

Several studies have shown that phototherapy effectiveness depends on the applied UVR dose. In areas protected from solar radiation, slower response to this therapy is observed. As for the patient's phototype, it seems to have no influence on treatment response.^41^

Several phototherapy modalities can be used in the treatment of sclerodermas, such as PUVA, UVA1 and NB-UVB. Topical PUVA can be used in the localized forms and systemic PUVA in generalized ones. NB-UVB is a viable option for scleroderma treatment, especially for lesions in the inflammatory phase, with superficial sclerosis. The preference, however, is for UVA1 radiation, as it shows deeper penetration into the dermis and the fact that there is a larger number of studies demonstrating its effectiveness.^10^, ^41^

### Atopic dermatitis

Atopic dermatitis (AD) is a common, recurrent, relapsing, chronic inflammatory disease. AD management includes avoiding triggering factors, trying to compensate for skin barrier defects, and maintaining anti-inflammatory therapy (topical corticosteroids and calcineurin inhibitors). When these first-line approaches are unsuccessful, systemic treatment or phototherapy should be considered.^42^, ^43^

Phototherapy has shown to be useful in the treatment of moderate to severe AD. The currently used modalities are NB-UVB, UVA1, PUVA and excimer laser/lamp.^42^, ^43^

Phototherapy has been classified as “Strength of Recommendation B” and “Level of Evidence II” in the treatment of AD. It is a second-line treatment, which should be reserved for cases in which behavioral and topical measures have failed, as numerous factors can limit its usefulness and effectiveness, including cost and access.^42^, ^43^

It acts by decreasing colonization by *Staphylococcus aureus*, improving the skin barrier function, reducing pruritus and tissue inflammation. Recent experimental studies have shown that its immunomodulatory effects include: decreased expression of IL-5, IL-13 and IL-31, as well as the induction of T-cell apoptosis and dendritic cell reduction.^42^, ^43^

The first modality of phototherapy used for AD treatment was broadband UVB, in 1970; however, due to its erythematogenic potential and low effectiveness, it fell into disuse. Morison et al. were the first to use PUVA for cases of refractory AD, with therapeutic success. Phototherapy can be used as monotherapy or in combination with emollients and steroids. Its use can reduce the need for topical or systemic immunosuppressants.^43^

The doses and frequency of PUVA sessions (topical or systemic) are similar to those used for psoriasis. This type of phototherapy is not the main choice of treatment for AD, because it does not show the best results and due to its mutagenic potential. Thus, it should be administered for short periods. The mechanism of action of PUVA phototherapy is yet to be fully understood.^43^

When treatment is carried out with UVA1, the average dose is the most indicated in most references. At this dose, the adverse effects are reduced and treatment becomes more tolerable. UVA1 has a more intense effect than UVB, so it is more appropriate for patients with acute AD. However, the first UVA1 lamps were expensive and required more space and adequate ventilation machinery, making them inaccessible in some centers. Regarding the mechanism of action of UVA1 therapy, the suppression of inflammatory cytokines, such as IL-5, IL-13 and IL-31 has been observed.^42^, ^43^

The best UVA1 dose in cases of AD exacerbation is a matter of debate. Retrospective studies have shown a reduction in SCORAD (Scoring Atopic Dermatitis/Atopic Dermatitis Severity Index) with both high and medium doses of UVA1. However, more prospective studies with larger samples are needed to establish the ideal dose.^44^, ^45^

NB-UVB has been used successfully since 1990 in AD. Currently, it is considered by most dermatologists as the first-line phototherapy modality for the treatment of AD, due to its availability, safety, easy administration, and effectiveness. This therapy reduced SCORAD and the need for topical corticosteroid use in several randomized studies. These benefits persisted for up to 6 months after the treatment regimen termination.^43^

The excimer laser is another treatment modality that can be used. Its use for 10 weeks has shown good results compared to clobetasol propionate. The excimer lamp in combination with emollients resulted in AD severity score improvement within a 4-week period.^43^

Considering the low accessibility to UVA1 devices when compared to other phototherapy modalities, NB-UVB provides the most successful and cost-effective treatment for patients with AD, with a proven improvement in the Health-Related Quality of Life (HRQoL) and the Dermatological Quality of Life Index (DLQI).^46^

### Photodermatoses

Phototherapy is an effective method to prevent seasonal outbreaks of photodermatoses. In general, the NB-UVB and PUVA doses are lower than the ones used for other dermatoses. It is a safe therapy, but it can cause skin rashes in a minority of patients, which does not limit treatment or worsen the prognosis. De Argila Fernández-Durán recommends the use of oral corticosteroids in the first days of treatment to prevent disease exacerbation.^47^

Polymorphic light eruption (PLE): NB-UVB has become the first-line therapy according to several authors because it is a practical method. It can be used even in the most severe cases. PUVA can also be considered.^48^

Actinic prurigo: NB-UVB or PUVA are viable options in extensive cases or those refractory to other therapies.^48^

Hydroa vacciniforme: some reports have shown symptoms relief in patients with this photodermatosis, but in most cases, this disease is resistant to treatment.^49^

Chronic actinic dermatitis: phototherapy is considered a second-line option, reserved for patients with contraindications for systemic immunosuppression or as prophylaxis. In such cases, it is possible to choose treatment with low-dose PUVA alone or in combination with topical and oral corticosteroid therapy for a prolonged period. NB-UVB can also be used, as well as UVA1 radiation.^49^

Solar urticaria: phototherapy can be used to prevent future crises since PUVA or NB-UVB induce phototolerance. In view of the risk of anaphylaxis, the minimum urticarial dose with the radiation intended for use should be tested, before starting the treatment. Additionally, the concomitant use of antihistamines is recommended.^49^

### Pityriasis Lichenoides

Pityriasis lichenoides chronica (PLC) is an uncommon dermatosis, of unknown etiology, for which phototherapy is one of the main treatments, particularly in the most extensive disease. There have been studies that corroborated the use of PUVA, NB-UVB and broadband UVB for this disease. NB-UVB is an effective treatment for the diffuse and chronic forms.^50^

There are small studies reporting the high effectiveness of treatment with NB-UVB, broadband UVB and PUVA. Despite the satisfactory results, due to the few cases included in these studies, it is not possible to draw a precise conclusion.^51^

## Other phototherapy indications

### Uremic pruritus

The mechanism of action of phototherapy in reducing the pruritus is unclear. NB-UVB decreases the production of IL-2, a cytokine related to pruritus, induces apoptosis of dermal mast cells and reduces the release of neuropeptides, such as substance P.^51^ Recent studies have shown that NB-UVB phototherapy can be considered an effective therapeutic option in the treatment of uremic pruritus.^52^

### Polycythemia vera

Pruritus is the most common symptom of polycythemia vera (PCV). Although its pathogenesis is not understood, it is believed that platelet and erythrocyte overproduction play a central role. The platelets aggregated in the skin vessels store and release prostaglandins and serotonin, both of which are involved in pruritus. There are studies evaluating the effectiveness of both NB-UVB and broadband UVB, as well as PUVA in these cases. Most studies included a small number of patients.^53^

NB-UVB phototherapy has shown a good risk/benefit ratio in the treatment of polycythemia vera-associated pruritus. However, further studies are needed to determine the ideal therapeutic regimen.^53^

### Prurigo nodularis

The excimer laser has been reported in the literature as a treatment modality that resulted in prurigo nodularis improvement. Larger investigations with long-term follow-up are needed to fully support its use.^54^

### Graft-versus-host disease

Graft-versus-host disease (GVHD) represents a complex immune response involving several organs. The disease occurs mainly in allogeneic hematopoietic stem-cell transplantation. The first-line treatment is carried out with high-dose corticosteroids, alone or in combination with other immunosuppressants, showing numerous side effects and increasing the risk of infections. In this sense, phototherapy plays an important role, as the treatment is directed at the skin, with few side effects.^55^

Its mechanism of action in these cases remains unknown, but apoptosis, antiproliferative and immunomodulatory effects seem to be involved. NB-UVB and UVA1 have been used more frequently in GVHD treatment, due to the safety of the methods and the important clinical response. One of the great advantages of associating phototherapy in the treatment is that it allows the reduction of corticosteroid doses.^56^

The following parameters must be considered when choosing the treatment modality: type, extent, and depth of the lesions, the possible involvement of other organs and use of concomitant medication. The treatment regimen is usually performed at lower doses than for other diseases. Phototherapy is effective in the treatment of both the acute and chronic phases, and in the prevention of graft-versus-host disease in adults and children.^56^

### Phototherapy and HIV

UVR is known to suppress the immune system and modify cytokine patterns. Moreover, exposure to UV rays is likely to increase viral replication. The main concern is the phototherapy indication in the early/intermediate disease stages when the patient still has a detectable viral load.^57^

There are some conditions that occur in HIV-infected patients which respond well to UVR use, such as psoriasis, eosinophilic folliculitis, eczema, and pruritus.^58^ The choice of UVR treatment should evaluate items such as skin lesion responsiveness and photosensitivity caused by some antiretrovirals.^57^

The risk-benefit ratio varies depending on the HIV disease stage. Existing data in advanced-stage patients suggest that treatment with NB-UVB or PUVA is not associated with clinical deterioration in the short term. In patients with an undetectable viral load, phototherapy may be considered.^58^ It should be noted that phototherapy seems to worsen the prognosis of patients with Kaposi's sarcoma and, therefore, this modality is contraindicated for these patients.^59^

### Phototherapy and carcinogenesis

Based on the mechanisms of action discussed in this review article, it is possible that UV radiation may have mutagenic potential. The reason for this concern is that workers exposed to sunlight have a higher incidence of melanoma and non-melanoma skin cancer, especially individuals with a low phototype.^9^

Studies have shown that PUVA induces cutaneous oncogenesis.^2^ A 10-fold increase in the risk of squamous cell carcinoma (SCC) has been reported when more than 150 treatments were performed (or a maximum cumulative dose of 1000–1500 J/cm). There is also scientific evidence of an increase in actinic keratoses.^4^, ^26^, ^60^

The risk of BCC, even in patients who received high doses of PUVA, is lower than that of SCC. The increased risk of melanoma after PUVA treatment is manifested 15 years after starting of the therapy.^60^

Although there is abundant evidence for PUVA dose-related skin cancer risk, studies investigating the risk of photocarcinogenesis with NB-UVB and UVA1 are limited to retrospective studies and case reports. According to the available evidence, NB-UVB seems, in general, to be the safest phototherapy modality.^60^

It should be noted that the combination of phototherapy and cyclosporine (including a history of cyclosporine use) should be avoided, considering the increased carcinogenic potential.^9^

## Other adverse effects

The adverse effects can be short-term or long-term ones. The most common acute adverse effect is erythema. If it is caused by PUVA, it appears between 48 and 72 hours after exposure and is usually prolonged. The erythema caused by UVB radiation occurs early, within the first 24 hours after exposure. If the erythema is mild and asymptomatic, the last dose used should be maintained. If there is severe erythema or if it is associated with pain, the treatment should be discontinued until the condition improves.^26^

In the long term, skin photoaging is an adverse effect inherent to all forms of phototherapy, being more intense with UVA, as it reaches deeper layers of the dermis. It is known that the lower the phototype, the greater the susceptibility to photoaging.^26^ Skin pigmentation changes also occur, with the formation of solar lentigines, both with PUVA and NB-UVB.

Pruritus may occur as a side effect. There are 2 types, one that depends on cutaneous xerosis and improves with emollient use and another with an idiopathic cause, which is rare, contraindicating the continuation of treatment.^4^, ^26^

Regarding the PUVA modality, gastrointestinal intolerance can occur with 8-MOP, usually being dose-dependent. The use of antiemetics and administration of psoralen after food intake attenuates this effect. Occasional symptoms include vertigo or headache. Intolerance reactions are specific to oral 8-MOP and can be prevented by replacing it with 5-MOP.^4^, ^26^

With PUVA, it is important to assess the risk of developing cataracts, and eye protection must be used during the session and for 12 hours after treatment, as 8-MOP can be detected in the lens up to 12 h after ingestion. A rare occurrence during treatment with this modality is acral blisters, which can develop in patients exposed to severe mechanical stress due to loosening of the dermo-epidermal junction.^4^, ^26^

The side effects of phototherapy rarely lead to its contraindication, but it may happen. The risk-benefit ratio for each case should be assessed and phototherapy, whenever possible, should be considered, since its benefits frequently outweigh the risks.

## Financial support

None declared.

## Authors' contributions

Norami de Moura Barros: Approval of the final version of the manuscript; design and planning of the study; drafting and editing of the manuscript; critical review of the literature; critical review of the manuscript.

Lissiê Lunardi Sbroglio: Approval of the final version of the manuscript; drafting and editing of the manuscript; collection, analysis, and interpretation of data; critical review of the literature.

Maria de Oliveira Buffara: Approval of the final version of the manuscript; drafting and editing of the manuscript; collection, analysis, and interpretation of data; critical review of the literature.

Jessica Lana Conceição e Silva Baka: Approval of the final version of the manuscript; drafting and editing of the manuscript; collection, analysis, and interpretation of data; critical review of the literature.

Allen de Souza Pessoa: design and planning of the study; critical review of the manuscript.

Luna Azulay-Abulafia: Approval of the final version of the manuscript; design and planning of the study; drafting and editing of the manuscript; effective participation in research orientation; critical review of the manuscript.

## Conflicts of interest

None declared.
